# Vaginal Ovariotomy

**Published:** 1876-12

**Authors:** 


### BIBLIOGRAPHICAL.

Vaginal Ovariotomy. By Clifton E. Wing, M.D., Boston. A re-print from the Boston Medical and Surgical Journal, November, 1876.

This pamphlet of seven pages describes an operation made for the removal of a diseased ovary, through the vagina. The safety of this method compared with the abdominal operation is discussed, and reference also made to the removal of healthy ovaries for the cure of neurotic and uterine diseases. The author hesitates to condemn this practice outright, but says: "Anxiety on the part of specialists to perform great or rare operations whenever a possible chance offers is unfortunate, as nothing tends more to produce a feeling of distrust of the specialty itself among the members of the profession." Again, he quotes from Allingham and others: 'It must be conceded that to obviate by judicious treatment the necessity for an operation, is more meritorious than to perform it well when required; and the fact that we are obliged in certain cases to resort to the removal of the ovaries to help our patients, is rather a reproach to medicine.' After alluding to the serious nature of the operation, the author refers to cases in which it was done without any benefit in the disease for which the females were unsexed. He declares that "in New York, where the operation has been done a number of times, the results, according to Dr. Munde, are not such as will probably lead to its abuse by repetition."

